# Hyperglycaemia-induced reciprocal changes in miR-30c and PAI-1 expression in platelets

**DOI:** 10.1038/srep36687

**Published:** 2016-11-07

**Authors:** Mao Luo, Rong Li, Meiping Ren, Ni Chen, Xin Deng, Xiaoyong Tan, Yongjie Li, Min Zeng, Yan Yang, Qin Wan, Jianbo Wu

**Affiliations:** 1 Drug Discovery Research Center, Southwest Medical University, Luzhou, Sichuan, China; 2 Laboratory for Cardiovascular Pharmacology of department of Pharmacology, the School of Pharmacy, Southwest Medical University, Luzhou, Sichuan, China; 3 Dalton Cardiovascular Research Center, University of Missouri, Columbia, MO, USA.; 4 Department of Endocrinology, the First Affiliated Hospital of Southwest Medical University, Luzhou, Sichuan, China

## Abstract

Type 2 diabetic mellitus (DM2) is associated with accelerated thrombotic complications and is characterized by high levels of plasminogen activator inhibitor-1 (PAI-1). Recent studies show that human platelets have high levels of miR-30c and synthesize considerable active PAI-1. The underlying mechanism of how PAI-1 expression is upregulated in DM2 is poorly understood. We now report that hyperglycaemia-induced repression of miR-30c increases PAI-1 expression and thrombus formation in DM2. Bioinformatic analysis and identification of miRNA targets were assessed using luciferase assays, quantitative real-time PCR and western blots *in* *vitro* and *in vivo*. The changes in miR-30c and PAI-1 levels were identified in platelets from healthy and diabetic individuals. We found that miR-30c directly targeted the 3′ UTR of PAI-1 and negatively regulated its expression. miR-30c was negatively correlated with glucose and HbA1c levels in DM2. In HFD-fed diabetic mice, increasing miR-30c expression by lenti-miR-30c significantly decreased the PAI-1 expression and prolonged the time to occlusion in an arterial thrombosis model. Platelet depletion/reinfusion experiments generating mice with selective ablation of PAI-1 demonstrate a major contribution by platelet-derived PAI-1 in the treatment of lenti-miR-30c to thrombus formation. These results provide important implications regarding the regulation of fibrinolysis by platelet miRNA under diabetic mellitus.

Patients with DM2 exhibit a 2- to 4- fold increase in risk for thrombotic complications, with cerebrovascular and severe peripheral vascular disease constituting leading causes of death in these patients^1^,^2^,^3^. In regard to underlying pathophysiological mechanisms, the activation of platelets appears to play a key role in the process^1^,^2^. The excessive activation and aggregation of platelets leads to exaggerated thrombus formation^4^,^5^,^6^. Consistent with this, platelet hyperaggregability appears to be involved in the pathogenesis of enhanced arterial thrombosis in DM2^5^,^7^. Importantly, platelets contain mRNAs and mRNA splicing machinery and translate mRNA into proteins relevant to haemostasis and inflammation^8^,^9^.

PAI-1 acts as the main circulating inhibitor of plasminogen activation in the fibrinolytic system^10^,^11^. In DM2, elevated levels of PAI-1 lead to impaired fibrinolytic function and have been associated with a greater risk of cardiovascular disease^12^,^13^. Recent studies show that platelets are capable of synthesizing large amounts of active PAI-1, which could be a mechanism by which platelets contribute to blood clot stabilization^14^,^15^. Of further relevance, some studies have noted that decreased insulin sensitivity is independently associated with higher PAI-1 or fibrinogen levels in platelets in DM2^16^,^17^.

MicroRNAs (miRNAs) act as important molecular biomarkers for haematologic disease and platelet reactivity and coordinate reactivity to the pathophysiological status, such as haemostasis and inflammation^3^,^8^,^18^,^19^,^20^,^21^,^22^,^23^. Among all miRNAs, the miR-30 family consists of five members (miR-30a, miR-30b, miR-30c, miR-30d, and miR-30e) that are highly conserved between human and mouse^24^,^25^, and has been widely investigated in a variety of human diseases^24^,^25^,^26^,^27^. Recent studies have shown that miR-30c targets PAI-1 in human cells^21^,^28^. In the present study, we found by comparing platelet genome-wide miRNA profile data from other studies, that miR-30c was expressed in platelets^20^,^29^,^30^,^31^. To date, however, these observations have received little or no attention from other researchers. In this study, using purified human platelets, we demonstrated the existence of a miR30c-targeted PAI-1 pathway in platelets of the cardiovascular system. The potential roles for miR-30c modulation of thrombus formation in DM2 and the mechanisms involved were then investigated.

## Methods

### Subjects

The patient study was conducted in accordance with the Declaration of Helsinki, and the study protocol was approved by the Ethics Committee of Affiliated Hospital of Southwest Medical University. All patients provided written informed consent. 174 research subjects were Han Chinese individuals who received oral glucose tolerance test (OGTT) and other medical health checkups. This study comprised four groups, including 50 healthy subjects (control), 50 pre-DM (pre-diabetic mellitus) subjects, 40 NCDM (non-complicated diabetic) subjects and 34 DM-CHD (diabetes mellitus type 2-coronary heart disease) subjects. The research subjects were not allowed to take aspirin, atorvastatin or nonsteroidal anti-inflammatory drugs 10 days prior to the investigation. Additional subject characteristics are detailed in “Supplementary Table S1”.

### Bioinformatic analysis

We searched for putative miRNAs that target the 3′ UTR of the PAI-1 mRNA using Targetscan human software^32^. The results were confirmed using different types of software, including miRanda^33^, MicroRNA.org^34^ and Microcosm^35^. All candidate miRNAs were then compared with the human platelet miRNA sequencing data. The evolutionary conservation was studied using MEME software (http://meme.nbcr.net/meme/tools/meme).

### Cell culture

The megakaryocyte cell line MEG-01 (ATCC, CRL-2021) was used to analyse gene expression in platelets as previously reported^20^,^30^.

### Leukocyte-depleted platelet preparation

Preparations of leukocyte-depleted platelets (LDP), platelet-rich plasma (PRP) and platelet-poor plasma (PPP) were performed as previously described^20^,^30^,^31^. Leukocyte depletion was performed by negative selection, using CD45^+^ MicroBeads (Miltenyi Biotec) according to the manufacturer’s instructions. To quantify levels of leukocyte contamination, we characterized the purified preparations by RT-PCR amplification of GPIIb and CD45 mRNA while also performing cell counts by haemocytometer.

### miR-30c and its target gene PAI-1 quantitative RT-PCR assay

Total RNA extraction was performed from all LDPs and MEG-01 cells with TRIzol (Invitrogen). Validation of the expression of the sequence-specific miR-30c was determined using quantitative stem-loop qRT-PCR using the NCode^TM^ miRNA First-Strand cDNA Synthesis Kit (Invitrogen). Validation of its target gene PAI-1 was performed using qRT-PCR using the M-MLV Reverse Transcription Kit (Promega, USA). U6 and 18S rRNA were used as internal controls for normalization. Gene expression was quantified as described previously^3^,^36^. All primers are listed in Supplementary Table S2.

### 3′ UTR luciferase reporter gene assay

A fragment of the PAI-1 mRNA 3′ UTR containing the putative or mutated miR-30c binding site was amplified by RT-PCR from MEG-01 cell total RNA. The products were inserted into the XhoI and NotI restriction sites of thepsi-CHECK2^TM^ vectors (Promega) downstream from the Renilla luciferase coding sequence. The constructs were cotransfected with miR-30c mimic, inhibitor and control oligo into HEK 293 cells using Lipofectamine 2000 (Invitrogen). Cells were harvested after 48 hours of transfection, and firefly luciferase activity was measured using the Dual-Luciferase Reporter Assay System Kit (Promega, E1910) and the luminometer Orion II (Berthold).

### Western blotting and ELISA analysis

Proteins were extracted, and the total protein concentration was determined by the BCA protein assay kit (Pierce). PAI-1 protein levels were evaluated by 4–20% SDS-PAGE with a Trans-Blot Semi-Dry transfer cell (Bio-Rad). After blocking, membranes were incubated with rabbit or mouse IgG which raised against human PAI-1. The horseradish-peroxidase (HRP)-conjugated goat IgG raised against rabbit or mouse IgG (Santa Cruz Biotechnology) was used as the secondary antibody. Blots were developed with ECL substrate (Pierce). PAI-1 antigen from LDPs or PRPs was measured using the human or mouse PAI-1 total antigen assay ELISA kit (Molecular Innovations).

### Assessing miR-30c over-expression or knockdown of PAI-1

miR-30c mimic, a double-stranded RNA molecule (numbered as miR10000244), miR-30c inhibitor, a single-stranded RNA molecule (numbered as miR20000244), or its negative control cel-miR-239b-5p (NC) are obtained from RiboBio (Guangzhou, Guangdong, China). MEG-01 cells (2 × 10^5^) were seeded in 24-well plates and co-transfected with miR-30c mimic (30 nM), inhibitor (30 nM) and NC control oligo (30 nM) using Lipofectamine 2000 (Invitrogen). All experiments were performed in triplicate. After 48 h, cells were harvested and the expression levels of PAI- 1 mRNA or protein were detected by qRT-PCR and Western blotting as described above.

### Animals

Male mice, 12–24 weeks old, were used. As a model for DM2, the leptin receptor deficient *db/db* mouse and its non-diabetic (normoglycemic and normolipidemic) heterozygote control (Db/db) were used. Studies involving *db/db* and Db/db mice were conducted under a protocol approved by the Animal Care and Use Committee, University of Missouri. The high-fat diet fed DM2 mouse model was produced as described previously^37^,^38^. C57BL/6J-congenic PAI-1-deficient (*Pai-1*^−/−^) mice were a gift from Dr. Peter Carmeliet, University of Leuven, Leuven, Belgium^39^. Protocols for animal use were reviewed and approved by the Animal Care Committee of Southwest Medical University in accordance with Institutional Animal Care and Use Committee guidelines.

### Platelet depletion, isolation and transfusion

To deplete endogenous platelets, mice were injected intraperitoneally with 2.5 μg/g mouse of platelet-depleting antibody (polyclonal anti-mouse GPIbα rat IgG, Emfret Analytics). Following injection of GPIbα antibody or control IgG, platelets were counted at appropriate time-points.

Both HFD-*Pai-1*^−/−^ and HFD-WT mice were anesthetized with ketamine/xylazine (intraperitoneal injection), and whole blood was collected from the inferior vena cava using a 1 mL syringe containing 0.1 mL sodium citrate anticoagulant. PRP was obtained from whole blood by centrifugation at 260 g, supernatant was collected after each centrifugation. The PRP was then centrifuged at 740 g for 10 minutes. The pellet containing platelets was then re-suspended in 1 mL of Phosphate Buffered Saline (Sigma Aldrich) and allowed to sit for 30 minutes, and the platelets were counted with a haemocytometer. Platelets from the suspension were then diluted with normal saline to 1.56 × 10^9^ platelets in 1.2 mL, and 0.2 mL of suspension transfused via tail vein into each recipient at 1 day after the injection of GPIbα antibody.

### Mouse model of high-fat diet-induced PAI-1 over-expression

C57BL/6J or *Pai-1*^−/−^mice were divided into two groups, which were fed either a high-fat chow diet (HFD) (D12451; Research Diet, New Brunswick, NJ) or a normal chow diet (NCD) for up to 14 weeks^37^,^38^. Food intake and body weight were measured once a week, and blood glucose levels were measured from tail vein blood samples using an automatic glucometer (Accu-Check; Roche Diagnostics, Mannheim, Germany).

### Carotid artery thrombosis model

Carotid artery thrombosis was created using topical FeCl_3_ as described previously^40^,^41^. The flow was monitored continuously from the onset of injury until stable occlusion occurred (defined as no flow for 25 minutes) by the Color Laser Doppler Image scanner (Moor LDI, Moor Instruments Ltd).

### Lenti-miR-30c injection *in vivo*

In order to generate plvx-shRNA2 + miR-30c recombinant plasmids, the amplification of miR-30c was performed using two specified synthetic DNA primers (Sangon Biotech, Shanghai, China) and the KOD plus neo DNA Polymerase kit (Toyobo) according to the manufacturer’s instructions, respectively (mir-30c *Bam*H1 forward: 5′CGCGGATCCACCATGTTGTAGTGTGTGTAAACATCCTACACTCTCAGCTGTGAGCTCAAGGTGG3′, and mir-30c *Eco*R1 reverse: 5′CCGGAATTCTCCATGGCAGAAGGA GTAAACAACCCTCTCCCAGCCACCTTGAGCTCACAGCTG3′). The PCR products were cloned into the *Bam*H1 and *Eco*R1 restriction sites of the plvx-shRNA2 vectors (Clontech), and then the transfer vector plasmids were purified, recovered, sequenced and identified. Lentivirus production was performed as described^42^,^43^. Briefly, 293T cells were transfected with the lentiviral constructs, VSV-G and p∆8.9 vectors to produce lentiviral particles. Particles were collected 48 h after transfection by 0.45 μm filtration of conditioned medium. The cells were mixed with filtered viral supernatant and polybrene (Sigma) and then placed at 37 °C overnight, and replaced with growth medium for 24 h. Selection with puromycin (Sigma) was started at 48 h after lentivirus transduction. Finally, the production of lenti-miR-30c droplets was adjusted to 1 × 10^8^ TU/ml.

HFD fed C57BL/6J mice were treated as described reported^37^,^38^. Age-matched male mice fed a normal chow diet served as controls. A total of 200 μl (1 × 10^7^ TU/ml, labelling with green fluorescent) of lenti-miR30c and lenti-NC were directly injected into the tail vein of all control and high-fat diet-fed C57BL/6J mice. After four days, the carotid artery injury was performed and the flow was monitored as described above. Time to occlusion was defined as the interval between the initiation of vascular injury and the onset of stable occlusion. To further determine the influence of thrombus formation and PAI-1 regulation after FeCl_3_ administration, vessels were clamped and the mice were euthanized with an overdose of anesthesia. The blood was collected, and LDPs were prepared. The gene expression levels of miR-30c and PAI-1 in LDPs were measured by qRT-PCR, and the PAI-1 total antigen was determined by ELISA as described above.

### Statistical Analysis

Data are expressed as the mean ± SEM. One-way analysis of variance with pairwise multiple comparisons, Student’s t-test, the log-rank test and SPSS Statistics (version 20.0) were used, as appropriate.

## Results

### Human platelets contain miR-30c and PAI-1

**Figure 1:**
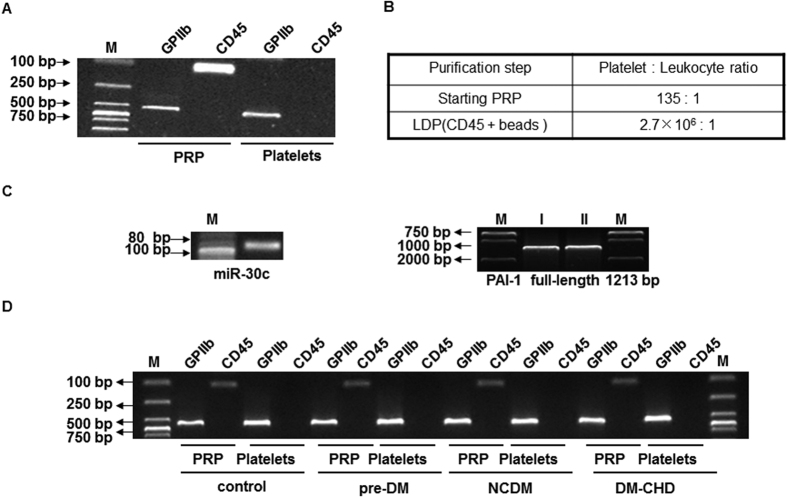
Human platelets contain miR-30c and PAI-1. (**A**) Healthy subject pooled LDP was prepared using CD45^+^ beads and then characterized using RT-PCR amplification of GPIIb and CD45 mRNAs. (**B**) Haemocytometer counts verifying successful leukocyte depletion. (**C**) Validation of sequence-specific human miR-30c and PAI-1 in LDP cDNA libraries by reverse transcription-PCR and agarose gel electrophoresis. I and II are experimental replicates. In the left panel of (C), “M” represents as 20 bp DNA Ladder Marker, “miR-30c” represents as human miR-30c. In the right panel of (C), “M” represents as DL2000 DNA marker. (**D**) LDP preparation and characterization by RT-PCR amplification of GPIIb and CD45 mRNA and agarose gel electrophoresis analysis in healthy subjects and patients with pre-DM, NCDM and DM-CHD.

Previous studies reported that platelets contain miR-30c, and high expression levels were found by comparing the genome-wide miRNA profile data in platelets^20^,^29^,^30^,^31^. We confirmed that miR-30c (MIMAT0000244) and PAI-1 (NM_000602.3) mRNA were found in the small/total RNA libraries of LDPs by PCR assay from healthy individuals. First, we assessed the LDP preparations by RT-PCR amplification of the leukocyte marker CD45 and the platelet-specific gene GPIIb. The cycle threshold of 37–40 indicated successful leukocyte depletion (Fig. 1A), which was further confirmed by haemocytometer counting (Fig. 1B). Next, we detected miR-30c and full-length PAI-1 mRNA by reverse transcription-PCR in LDPs from healthy individuals (Fig. 1C), indicating that platelets contain miR-30c and PAI-1. LDP isolated from healthy individuals and patients with pre-DM, NCDM and DM-CHD were measured as described above (Fig. 1D), indicating the marked and successful depletion of leukocytes from the starting PRP.

### Prediction analysis

Predictions of miR-30c target genes were performed as described above in “Methods-Bioinformatic analysis”, and the results provided information regarding target site accessibility. As shown in Supplementary Figure S1A, there is only a single predicted miR-30c target site (643 bp–669 bp) in the PAI-1 mRNA 3′ UTR based on good complementarity (ΔG°~ −25.67 kcal/mol). The extent of evolutionary conservation identified using MEME software included *Homo sapiens*, *Macaca mulatta*, *Bos taurus*, *Mus musculus* and *Rattus norvegicus* (Supplementary Figure S1B), indicating a high degree of site conservation among different mammalian species.

### Reciprocal changes of platelet miR-30c and PAI-1 levels in DM2

**Figure 2:**
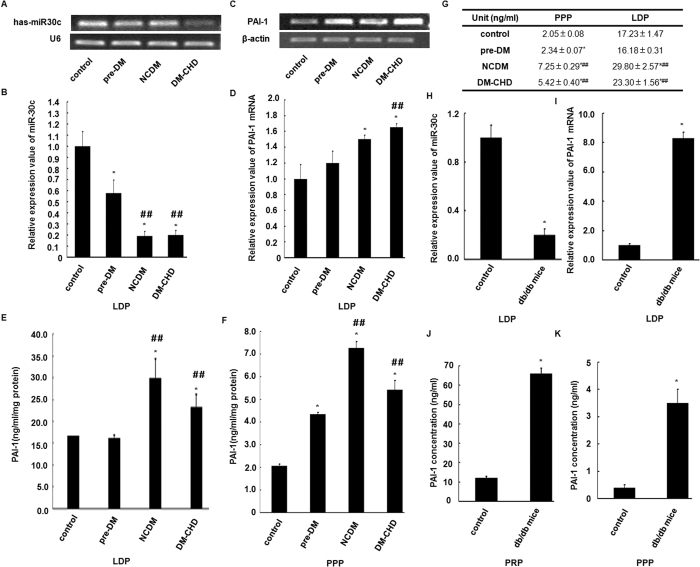
Reciprocal changes of platelet-derived miR-30c and PAI-1 levels in DM2. (**A,B**) Reciprocal changes in expression of miR-30c and its target gene PAI-1 mRNA levels were confirmed through qRT-PCR analysis in healthy subjects (control, n = 50), pre-diabetic mellitus subjects (pre-DM, n = 50), non-complicated diabetic subjects (NCDM, n = 40) and diabetes mellitus type 2-coronary heart diabetic subjects (DM-CHD, n = 34) LDPs. The data were normalized to U6 RNA (for miR-30c) and 18S rRNA (for PAI-1) in each sample. (**E**,**F**) PAI-1 antigen levels were determined using ELISA following a BCA assay for LDP and PPP in healthy, pre-DM, NCDM and DM-CHD subjects. (**G,H**) Down- and up-regulation of miR-30c and PAI-1mRNA levels were detected by qRT-PCR in *db/db* mice LDPs compared to control mice. (**I,J**) PAI-1 antigen levels were determined by ELISA following a BCA assay in PRP and PPP of *db/db* mice *vs*. controls. All data are presented as the mean number per section ±SEM. *p < 0.05 *vs*. controls. ^##^*p* < 0.05 NCDM and DM-CHD subjects *vs*. pre-DM subjects.

MiR-30c and PAI-1 mRNA levels in LDPs were analysed by qRT-PCR and compared with the levels of endogenous genes, U6 and 18S rRNA. As shown in Fig. 2A,B, miR-30c levels progressively decreased in LDP samples from patients classified as pre-DM, NCDM and DM-CHD. The lowest expression was found in NCDM and DM-CHD, with a significant decrease (5-fold) compared with healthy individuals.

Next, we identified changes in the PAI-1 mRNA level. The PAI-1 mRNA expression levels were up-regulated and significantly greater in the DM-CHD subjects compared with other groups (Fig. 2C,D). Furthermore, we estimated the total amount of PAI-1 protein antigen by ELISA in LDP and PPP samples. As shown in Fig. 2E,F, the average amount of PAI-1 antigen increased 4- to 8- fold in LDP compared with PPP. Higher levels of PAI-1 protein were found in NCDM and DM-CHD compared with pre-DM and control.

To further determine the reciprocal changes of miR-30c and PAI-1 in DM2, we analyzed miR-30c and PAI-1 levels in the LDPs, PRP and PPP from *db/db* and corresponding control mice. Similarly, there was also significantly lower expression of miR-30c and higher expression of PAI-1 mRNA and protein in *db/db* mice compared with control mice (Fig. 2G–J). These results from an animal model are therefore consistent with there being reciprocal changes in platelet miR-30c and PAI-1 levels in DM2.

### PAI-1 is a direct target of miR-30c

**Figure 3:**
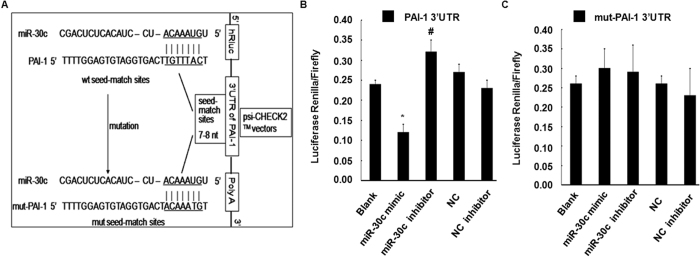
PAI-1 is a direct target of miR-30c. (**A**) Schematic representation of the PAI-1 3′ UTR luciferase reporter plasmid. The “seed sequences” and the point mutations in the seed sequences are underlined. (**B,C**) A miR-30c mimic (30 nmol/L), miR-30c inhibitor (30 nmol/L) or control oligo (30 nmol/L) was co-transfected with the psi-CHECK-2 wild-type or mutated PAI-1 3′ UTR sequence vectors in HEK293 cells. The relative luciferase activity is reported. All data are presented as the mean ±SEM of triplicate independent experiments. *p < 0.05, miR-30c mimic experimental *vs*. Blank and NC. ^#^*p* < 0.05, miR-30c inhibitor experimental *vs*. Blank and NC inhibitor.

To investigate the predicted interaction of miR-30c with PAI-1, the 3′ UTR of human PAI-1 containing the putative miR-30c binding sites was cloned into the psi-CHECK2^TM^ vector downstream of the Renilla luciferase coding sequence and co-transfected with miR-30c mimic, inhibitor or control oligo into HEK 293 cells. An empty vector was used as control (Fig. 3A). In the presence of the PAI-1 3′ UTR, the miR-30c mimic significantly decreased the relative luciferase activity to approximately 55% compared to co-transfection with miR-NC. The miR-30c inhibitor increased the relative luciferase activity to approximately 12% (Fig. 3B). Furthermore, to investigate whether the predicted miR-30c binding sites mediate the effect on PAI-1, miR-30c seed sequences binding to the PAI-1 mRNA 3′ UTR were mutated (Fig. 3A). The inhibitory effect of the miR-30c mimic and enhancement of the miR-30c inhibitor were indeed abrogated compared to co-transfection of control oligo with vector or empty vector (Fig. 3C). Thus, miR-30c modulated reporter gene expression through the PAI-1 mRNA 3′ UTR seed sequence and directly negatively regulated its expression.

### Platelet expressed miR-30c negatively regulates PAI-1 levels

**Figure 4:**
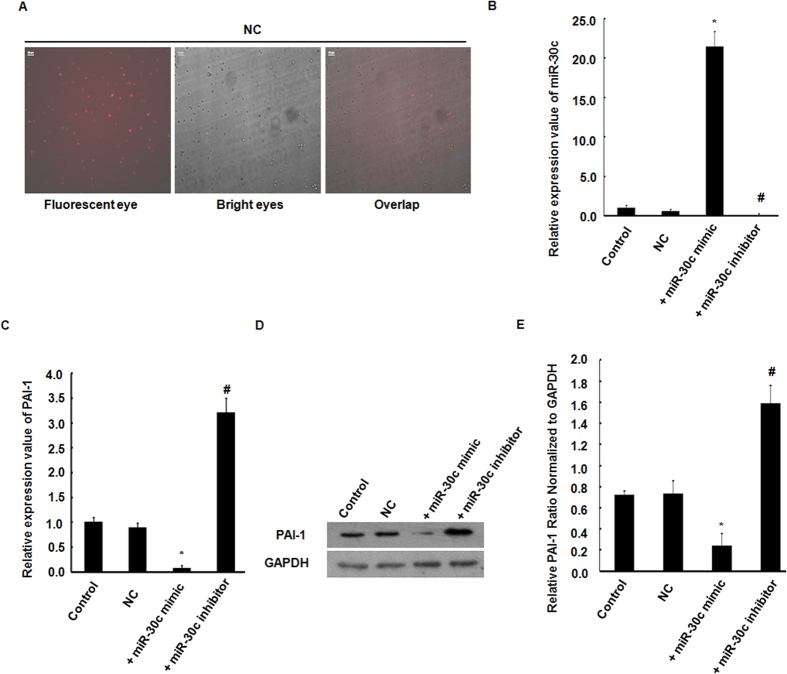
Platelet-derived miR-30c negatively regulates PAI-1 mRNA and protein levels. (**A**) Transfection efficiency was evaluated using fluorescence. Scale bars = 100 μm. (**B,C**) PAI-1 mRNA level swere significantly reduced by the over-expression of miR-30c in response to miR-30c mimic, and increased in response to miR-30c inhibitor, as determined using qRT-PCR after transfection in MEG-01 cells. (**D,E**) PAI-1 protein expression was down-regulated by the miR-30c mimic but up-regulated by the miR-30c inhibitor, as determined by Western blotting normalized to GAPDH. All data are the mean ± SEM. Densitometric analysis of 3 independent experiments. *p < 0.05, miR-30c mimic experimental *vs*. control and NC values. ^#^*p* < 0.05, miR-30c inhibitor experimental *vs*. control and NC.

We investigated a potential role of miR-30c as a mediator of PAI-1mRNA and protein levels in platelets by transfection with the miR-30c mimic or inhibitor in MEG-01 cells. A transfection efficiency of up to 60–70% of test negative control (NC) was evaluated by fluorescence (Fig. 4A). Transfection with miR-30c mimic significantly increased miR-30c gene expression (Fig. 4B) and significantly inhibited the expression levels of the PAI-1 mRNA and protein compared to a NC (Fig. 4C–E). In contrast, transfection with the miR-30c inhibitor showed a significant reduction in miR-30c expression and a significant increase in PAI-1 mRNA and protein levels when compared to a NC (Fig. 4B–E).

### miR-30c modulates thrombus formation *in vivo*

**Figure 5:**
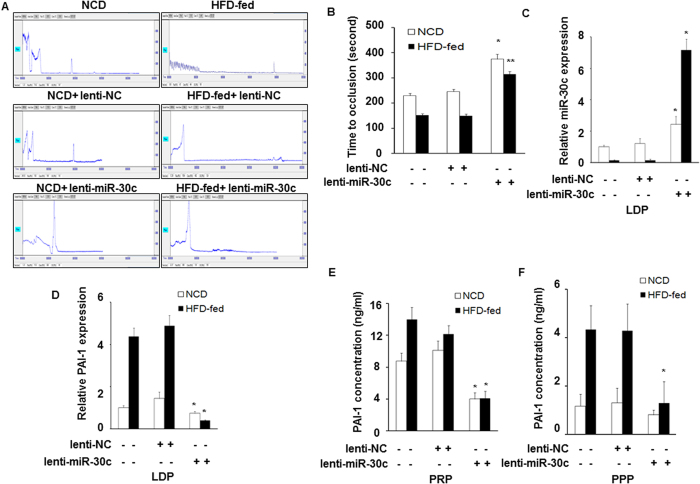
miR-30c modulates thrombus formation *in vivo*. (**A**,**B**) Carotid arterial occlusion after lenti-miR-30c injection in the FeCl_3_-induced injury mouse model. Carotid artery blood flow tracings collected using the Color Laser Doppler Image scanner are shown in the upper panel. The mean times to thrombotic occlusion of carotid arteries obtained from NCD-fed and HFD-fed mice (n = 8) are indicated in the lower panel. *p < 0.05, experimental *vs*. lenti-NC injection NCD-fed mice and *vs*. NCD-fed mice. **p < 0.05 *vs*. lenti-NC injection NC mice and *vs*. HFD mice. (**C,D**) The expression of miR-30c and PAI-1 mRNA levels were measured by qRT-PCR in LDPs from HFD-fed and NCD-fed mice after the lenti-miR-30c and lenti-NC injection. (**E,F**) PAI-1 antigen levels were determined by ELISA following a BCA assay in PRP and PPP. All data are presented as the mean number per section ± SEM. *p < 0.05, experimental *vs*. lenti-NC injection NCD-fed mice and *vs*. NCD-fed mice.

To assess the effect of miR-30c on the arterial thrombosis relevant to DM2, we fed mice a HFD for 14 weeks, which produced obesity and hyperglycaemia, and high plasma leptin levels (Supplementary Figure S2). An arterial thrombosis model was induced in fed mice by FeCl_3_ injury. After the lenti-miR-30c injection, the blood flow was recorded using the Color Laser Doppler Image scanner. The mean time to thrombotic occlusion in HFD-fed mice (152 ± 6.2 seconds; n = 8) was shorter than that of NCD-fed mice (230 ± 8.9 seconds; n = 8) (Fig. 5A,B). Following injection of lenti-miR-30c, the mean time to thrombotic occlusion in HFD-fed mice (315 ± 11.2 seconds; n = 8) or NCD-fed mice (375 ± 19.2 seconds; n = 8) dramatically increased compared with lenti-NC mice (lenti-NC-HFD-fed, 150 ± 7.5 seconds and lenti-NC-NCD-fed, 246 ± 9.7 seconds; n = 8) (Fig. 5A,B). These results suggest that miR-30c modulates arterial thrombus formation independently of diabetes.

### *In vivo* analysis of miR30c regulating PAI-1 in platelets

**Figure 6:**
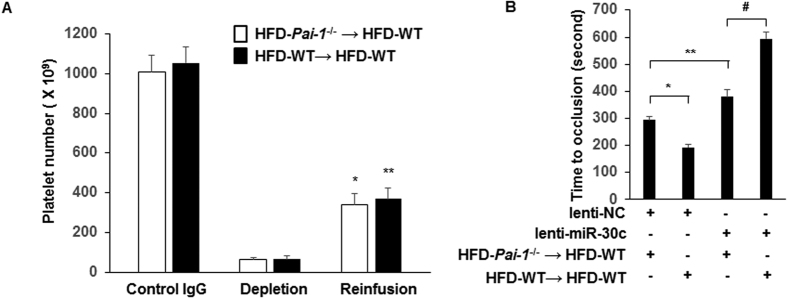
Platelet-derived miR-30c is critical for modulating arterial thrombosis. (**A**) Platelet numbers were counted in whole blood drawn from HFD-WT mice. Platelet depletion was achieved by injection of anti-mouse GPIbα rat IgG (*n = *11). *P < 0.05 vs. depleted HFD-*Pai-1*^−/−^ → HFD-WT mice; **P < 0.05 vs. depleted HFD-WT → HFD-WT mice. (**B**) Mean time to thrombotic occlusion of carotid arteries was measured from the 4 groups of mice generated by the platelet depletion/reinfusion as indicated in the figure. (n = 11/each group). *p < 0.05, lenti-NC injection HFD-WT → HFD-WT *vs*. lenti-NC injection HFD-*Pai-1*^−/−^ → HFD-WT mice; **p < 0.05 lenti-miR-30c injection HFD-WT → HFD-WT *vs*. lenti-NC injection HFD-WT → HFD-WT mice; ^#^*p* < 0.05, lenti-miR-30c injection HFD-WT → HFD-WT *vs*. lenti-miR-30c injection HFD-*Pai-1*^−/−^ → HFD-WT mice.

To investigate whether miR-30c negatively regulates PAI-1 levels *in vivo*, blood was collected following the studies of thrombus formation, and LDP, PRP, and PPP were prepared as described above. MiR-30c and PAI-1 mRNA levels in LDPs were determined by real-time PCR. As shown in Fig. 6C,D, miR-30c expression in HFD mice was significantly lower than in WT mice. After the lenti-miR-30c injection, miR-30c levels were significantly increased (50-fold) in HFD-fed mice compared with those of lenti-NC mice (Fig. 5C). In contrast, PAI-1 mRNA levels were significantly greater in HFD-fed mice than in NCD-fed mice. After the lenti-miR-30c injection, PAI-1 mRNA expression was significantly reduced (~90%) in HFD-fed mice compared to lenti-NC mice (Fig. 5D). Similarly, levels of PAI-1 protein in PRP or PPP were greater in HFD-fed mice than that in NCD-fed mice. PAI-1 protein levels in PRP or PPP were significantly reduced after injection with lenti-miR-30c in HFD-fed mice compared to lenti-NC mice (Fig. 5E,F).

### Platelet depletion/reinfusion procedure identifies miR-30c as a critical regulator of arterial thrombosis in DM2

To study the contribution of platelet miR-30c in thrombosis and PAI-1 levels, we used a platelet depletion/reinfusion model to examine arterial thrombosis in different groups (HFD-*Pai-1*^−/−^ → HFD-WT, HFD-WT → HFD-WT). Consistent with our previous report^41^, circulating platelets were successfully reduced by anti-mouse GPIb rat IgG by more than 90% compared with control IgG (Fig. 6A). Reinfusion of platelets from a donor mouse to thrombocytopenic mice successfully increased platelet counts 4.37- to 5.54- fold compared to platelet-depleted mice (Fig. 6A).

After the lenti-NC injection, mean time to occlusive thrombus formation in HFD-WT → HFD-WT group (193 ± 8 seconds, n = 11) was shorter than that of HFD-*Pai-1*^−/−^ → HFD-WT group (294 ± 12 seconds, n = 11, p < 0.05) (Fig. 6B), indicating that platelet-derived PAI-1 plays a key role in accelerating thrombosis formation. Furthermore, the treatment of lenti-miR-30c significantly inhibited thrombus formation in both the HFD-*Pai-1*^−/−^ → HFD-WT group (mean time to thrombotic occlusion increased to 379 ± 26 seconds, n = 11, *P* < 0.05) and the HFD-WT → HFD-WT group (mean time to thrombotic occlusion increased to 594 ± 26 seconds, n = 11, *P* < 0.05). As the mean time to occlusion was significantly prolonged by treatment of lenti-miR-30c compared to lenti-NC in HFD-*Pai-1*^−/−^ → HFD-WT group, this suggests a contribution PAI-1 from a source other than platelets, possibly vascular cells (i.e., endothelial and smooth muscle cells), to thrombus formation. Importantly, after the lenti-miR-30c, mean time to occlusion in HFD-WT → HFD-WT group was significantly longer than that of HFD-*Pai-1*^−/−^ → HFD-WT group. Noteworthy, a markedly prolonged occlusion time was observed between HFD-WT → HFD-WT to HFD-*Pai-1*^−/−^ → HFD-WT in the presence of lenti-miR-30c, when compared with HFD-*Pai-1*^−/−^ → HFD-WT in the presence or absence of lenti-miR-30c (3^rd^–4^th^ *Vs*. 1^st^–3^rd^ bar in Fig. 6B). Together, these findings suggest that platelet-derived PAI-1, regulated by miR-30c, plays a major role in the modulation of thrombosis formation in DM2.

## Discussion

Human platelets contain miRNAs, which perform important regulatory roles in platelet production and activation in a variety of thrombotic diseases^8^,^22^,^44^. Studies have revealed that DM2 is an important contributor to thrombotic disease burden, inducing dysfunctional platelets and affecting platelet miRNAs^22^,^44^, mRNAs and proteins^1^,^5^. However, the mechanisms by which platelet miRNAs regulate biological pathways in DM2 are still poorly understood^45^.

miRNAs play vital roles in the regulation of mRNA stabilization and degradation^21^,^46^. In this study, we successfully identified putative miRNAs that showed complementarity to target the 3′ UTR of the PAI-1 mRNA. The results revealed potentially conserved sites for approximately nine miRNA family candidates (miR-30c, miR-34a/c, miR-449b, miR-181, miR-301a, miR-421, miR-299-5p, miR-609 and miR-99a) in the PAI-1 mRNA 3′ UTR. All candidate miRNAs were compared with the published human platelet miRNA sequencing data and other published studies. We demonstrated that only miR-30c was especially enriched and jointly expressed with PAI-1 in platelets. The results also revealed that miR-30c had high complementarity and a high degree of species conservation with respect to binding sites within the 3′ UTR of the PAI-1 mRNA. Furthermore, we found that miR-30c could modulate reporter gene expression through the PAI-1 mRNA 3′ UTR seed sequence and directly negatively regulated its mRNA and protein expression in megakaryocytes, consistent with PAI-1 being a direct target of miR-30c and that platelet miR-30c negatively regulates PAI-1 levels.

PAI-1 is known for its role in regulating the balance between the fibrinolytic system and the thrombotic system^10^,^11^,^47^. Previous studies have shown that patients with DM2 have high levels of PAI-1, which acts as an independent thrombotic risk factor^13^,^16^. In this study, we found significantly lower expression of miR-30c and higher expression of PAI-1 mRNA and protein in patients with NCDM and DM-CHD compared with healthy subjects. The finding of reciprocal changes of platelet miR-30c and PAI-1 levels in PLT, PRP and PPP in subjects with DM2 leads to the hypothesis that decreased platelet miR-30c removes a normally inhibitory influence on PAI-1 levels and thus plays a significant role in thrombus formation. Several clinical studies have reported that circulating PAI-1 increases the risk of thrombosis, whereas inhibition of PAI-1 may have antithrombotic consequences^48^,^49^. Some studies also have reported that PAI-1 is a major determinant of the resistance of platelet-rich arterial thrombi to lysis, whereas inhibition or resistance to PAI-1 may enhance thrombolysis^48^,^50^. Using the FeCl_3_-induced arterial thrombosis model, we found that the carotid arterial time to occlusion for HFD-fed mice was shorter than that of mice fed a normal chow diet. To study the effect of miR-30c on thrombosis, lenti-miR30c and lenti-NC were directly injected into the tail vein in mice. Increased miR-30c expression by lenti-miR-30c injection significantly decreased the expression of PAI-1 mRNA and protein and prolonged the arterial time to occlusion in HFD-fed diabetic mice, thereby modulating arterial thrombus formation. This finding indicates that platelet miR-30c plays a key role in mediating arterial thrombosis and has antithrombotic consequences. Further support for this was provided by studies where platelets from WT or *Pai-1*^−/−^ mice were reinfused into mice depleted of platelets by serial injection of anti-mouse GPIbα rat IgG. In this situation, lenti-miR30c administration resulted in a significant inhibitory effect on thrombosis formation. These experiments further demonstrate that platelet-derived PAI-1, regulated by miR-30c, plays a critical role in modulating thrombosis in DM2. However, miR-30c has multiple targets including growth factors^26^, extracellular matrix proteins^51^, cytokine receptors^52^, transcription factors, and ADAM family members), and miR-30c is probably mediated by some upstream transcription factors including Spermatogenic leucine zipper protein 1 (Spz1), (sex determining region Y)-box 17 (SOX17), and Hepatocyte Nuclear Factor-3 Homologue 1 (HFH-1) etc., suggesting that additional experiments will be required to further define the upstream and downstream regulatory network of miR-30c on thrombosis formation in DM2 in future studies.

Prior studies have demonstrated that, in subjects with glucose tolerance, levels of PAI-1 increase significantly with increasing fasting blood glucose levels^53^,^54^. In this study, we found that subjects (DM-CHD) with the highest fasting blood glucose concentrations (FPG and 2 h FPG) by an OGTT had higher body mass index (BMI), total cholesterol (TC), triglyceride (TG), low-density lipoprotein cholesterol (LDL-C), waist circumference (WC), blood pressure (BP), and PAI-1 than subjects (control and pre-DM) with normal glucose concentrations (Table S1). High-density lipoprotein cholesterol (HDL-C) was lower with increasing levels of fasting blood glucose within the normal range. We also found that subjects with DM2 had the highest PAI-1 levels, with increasing levels of haemoglobin A1c (HbA1c) compared to healthy subjects (Supplementary Table S1). The increased level of PAI-1 correlated with increasing fasting glucose levels, HbA1c and other baseline characteristics in DM2, especially in subjects with DM-CHD. Further, we demonstrated that miR-30c was negatively related to glucose and HbA1c levels.

In conclusion, we described a novel regulatory mechanism of miR-30c regulating conserved target PAI-1 mRNA and protein expression by directly binding to the PAI-1 mRNA 3′ UTR seed sequence. Further, we found an apparent negative relationship between miR-30c levels and glucose and HbA1c levels in subjects with DM2. In experimental animal models subjected to high fat feeding, miR-30c modulates thrombus formation by regulating PAI-1 levels, furthermore, platelet depletion/reinfusion experiments generating mice with selective ablation of PAI-1 demonstrate a major contribution by platelet-derived PAI-1, which is regulated by miR-30c, plays a major role in the modulation of thrombosis formation in DM2, consistent with platelet miRNAs playing an important regulatory role for the fibrinolytic system in DM2. Overall, our present work demonstrates that miR-30c can be efficiently measured in platelets, and provides impetus for assessing the role of miR-30c as a novel biomarker for thrombotic complications of DM2. These findings support proceeding to additional preclinical studies involving novel miRNA-based therapeutic strategies against thrombosis formation in DM2.

## Additional Information

**How to cite this article**: Luo, M. *et al*. Hyperglycaemia-induced reciprocal changes in miR-30c and PAI-1 expression in platelets. *Sci. Rep*. **6**, 36687; doi: 10.1038/srep36687 (2016).

**Publisher’s note**: Springer Nature remains neutral with regard to jurisdictional claims in published maps and institutional affiliations.

## Supplementary Material

Supplementary Information
