# Supplementary material for: Hyperglycaemia-induced reciprocal changes in miR-30c and PAI-1 expression in platelets
Source: Sci Rep. 2016 Nov 7;6:36687. doi: 10.1038/srep36687 (PMC5098184; doi:10.1038/srep36687)

Supplementary Information for

Hyperglycaemia-induced reciprocal changes in miR-30c and PAI-1 expression in platelets

Mao Luo [1,2], Rong Li[1,2], Meiping Ren[1,2], Ni Chen[1,2], Xin Deng[1,2], Xiaoyong Tan[1,2], Yongjie Li[1,2], Min Zeng[1,2], Yan Yang [4], Qin Wan[3*], Jianbo Wu[1,2,4*]

[1] Drug Discovery Research Center, Southwest Medical University, Luzhou, Sichuan, China.
[2] Laboratory for Cardiovascular Pharmacology of department of Pharmacology, the School of Pharmacy, Southwest Medical University, Luzhou, Sichuan, China.
[3] Department of Endocrinology, the First Affiliated Hospital of Southwest Medical University, Luzhou, Sichuan, China.
[4] Dalton Cardiovascular Research Center, University of Missouri, Columbia, MO.

[*]Correspondence: Jianbo Wu, Division of Cardiovascular Medicine, University of Missouri, 5 Hospital Drive, CE344–DC095.00, Columbia, Missouri 65212. Email: wuji@missouri.edu, Phone: +86-830-4040, Fax: (573) 884-7743. Or to: Qin Wan, Department of Endocrinology, The Affiliated Hospital of Southwest Medical University, Luzhou 646000, China. Email: wanqin3@163.com.

Supporting Information

Supplementary Table S1. Baseline characteristics of the study population. Data are shown as the mean ± standard deviation. BMI, body mass index; TC, total cholesterol; TG, triglyceride; HDL-C, high-density lipoprotein cholesterol; LDL-C, low-density lipoprotein cholesterol; BP, blood pressure; WC, waist circumference; HbA1c, hemoglobin A1c.

Supplementary Table S2. Sequences of primers used in the study.

Supplementary Figure S1. PAI-1 is a predicted miR-30c target with high complementarity and evolutionary conservation. (A) Prediction of miR-30c targeting PAI-1 by bioinformatic analysis. Sequence conservation of the predicted miR-30c binding sites in the 3' UTR of PAI-1, with the miR-30c seed and seed matches highlighted in yellow. (B) The extent of evolutionary conservation of miR-30c in different species was analysed by MEME software.

Supplementary Figure S2. Diabetic mice induces obesity, hyperglycemia, and increased plasma leptin in mice. db/db mice metabolic changes, (A) body weight, (B) plasma glucose, (C) plasma leptin. (n=5/group); *P<0.05 vs. db/db group. Mice were fed high-fat diet (HFD) or normal diet (NCD) for 14 weeks, after which (D) body weight, (E) plasma glucose, (F) plasma insulin, and (G and H) plasma leptin were measured (n=5/group); *P<0.05 vs. NCD group, or lenti-NC injection HFD-*Pai-1*$^{-/-}$→HFD-WT mice; ***p*< 0.05 *vs*. lenti-NC injection HFD-WT→HFD-WT mice.

Supplementary Table S1. Baseline characteristics of the study population.

	Healthy controls	pre-DM	NCDM	DM-CHD
Number, n	50	50	40	34
Gender (male/female)	31/19	23/27	21/29	15/19
Age, years	52.2 ± 5.5	53.6 ±5.8	58.6 ± 6.2	61.3 ± 6.5
BMI, kg/m^2	23.6 ±2.8	25.2 ± 3.2	27.3 ± 4.2	28.5 ± 4.8
TC, mmol/L	5.0 ± 0.8	4.7 ± 0.9	6.1± 0.5	6.6± 0.9
TG, mmol/L	1.7 ± 0.4	1.6 ± 0.4	1.9± 0.6	2.3± 0.9
HDL-C, mmol/L	1.3 ± 0.1	1.3 ± 0.2	1.1 ± 0.1	1.1 ± 0.2
LDL-C, mmol/L	2.1 ± 0.5	2.2 ± 0.6	2.6 ± 0.8	2.8 ± 0.5
Current smoker (%)	32.0	36.0	35.0	44.1
BP (Systolic / diastolic),mmHg	108.6 ±11.2 /80.0 ± 4.0	115.5 ±11.5 /80.5 ± 4.5	131.6 ±20.2 /82.7 ± 8.66	139.2 ±24.3 /84.0 ± 11.2
WC, cm	79.6 ± 10.2	82.5 ± 9.6	104.5 ± 11.6	105.0 ± 13.2
Blood GLU (mmol/L) FPG	4.8±0.4	6.2±0.8	7.8±0.8	88.2±1.1
2h PG	6.3±0.8	8.1±1.2	12.2±1.4	13.5±1.5
HbA1c (%)	5.3± 0.2	5.8 ± 0.3	7.3 ± 0.5	7.5 ± 1.2

Supplemental Table S2. Sequences of primers used in the study.

	Primer	Sequences
LDP preparations measuring	Sense primer of CD45	5'- AACAGTGGAGAAAGGACACA-3'
	Antisense primer of CD45	5'- TGTGTCCAGAAAGGCAAAGC-3'
	Sense primer of GPIIb	5'- GAATCGCGATGTTGGTGAGC-3'
	Antisense primer of GPIIb	5'- GGTCACACTGCACCACAGTA-3'
Stem-loop Q-RT-PCR for miR-30c	Stem-loop RT primer of miR-30c	5'-CTCAACTGGTGTCGTGGAGTCGGCA ATTCAGTTGAGGCTGAGA -3'
	Sense primer of miR-30c	5'- ACACTCCAGCTGGGTGTAAACATCCTACACTC -3'
	Antisense primer of miR-30c	5'- CTCAACTGGTGTCGTGGA -3'
	Sense primer of reference gene U6	5'-CTCGCTTCGGCAGCACA-3'
	Antisense primer of reference gene U6	5'-AACGCTTCACGAATTTGCGT-3'
Real-time PCR for human PAI-1	Sense primer of PAI-1	5'- TTCAGGCTGACTTCACGAGT -3'
	Antisense primer of PAI-1	5'- CCAGATGAAGGCGTCTTTCC -3'
	Sense primer of reference gene18S rRNA	5'-CCTGGATACCGCAGCTAGGA-3'
	Antisense primer of reference gene 18S rRNA	5'-GCGGCGCAATACGAATGCCCC-3'
Vector construction	Sense primer of PAI-1 XhoIF	5'- CCGCTCGAGCCCTGGGGAAAGACGCCT -3'
	Antisense primer of PAI-1 NotIR	5'- ATAAGAATGCGGCCGCTTGACTGTCCTGACA

		TATTCTTCGTATTTA-3’
	Sense primer of mut-PAI-1	5’- ATTTTGGAGTGTAGGTGACTACAAATGTCATT GAAGCAGATTTCTGC-3’
	Antisense primer of mut-PAI-1	5’- GCAGAAATCTGCTTCAATGACATTTGTAGTCAC CTACACTCCAAAAT-3’
Lenti-miR-30c Vector construction	mir-30c Bam HIF	5’- CGCGGATCCACCATGTTGTAGTGTGTGTAAAC ATCCTACACTCTCAGCTGTGAGCTCAAGGTGG-3’
	mit-30c Eco RIR	5’-CCGGAATTCTCCATGGCAGAAGGAGTAAACAA CCCTCTCCCAGCCACCTTGAGCTCACAGC-3’
Real-time PCR for mice PAI-1	Sense primer of PAI-1	5’- GGACACCCTCAGCATGTTCA-3’
	Antisense primer of PAI-1	5’- TCTGATGAGTTCAGCATCCAAGAT-3’
	Sense primer of reference gene GAPDH	5’-TGCCCAGAACATCATCCCTG-3’
	Antisense primer of reference gene GAPDH	5’-TGAAGTCGCAGGAGACAACC-3’

Supplementary Figure S1.

A

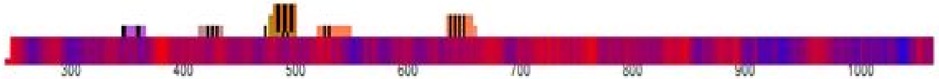


```
                                        hsa-miR-30c
                                        15.5003:643:5951
                                        cGACUCUCACAUC--CU--ACAAAUGu
                                         :| :||||||||  ||  |||||||
H.Sapiens ENST00000223095             UGGGUUAUUUUGGAGUGUAGGUGACUUGUUUACUCAUUGAAGCAGAUUU
M.Mulatta ENSMMUT00000013862          UGGGUUAUUUUGGAGUGUAGGUGACUUGUUUACUCAUUGAAGCAGAUUU
B.Taurus ENSBTAT00000019232           UAUAUUAUUUUAGAGUGUAGAUGACUUGUUUACUCAGAGAAGCAGGUUU
C.Familiaris ENSCAFT00000022067      -------------------------------------------------
M.Musculus ENSMUST00000041388         UAUGUUAUUUCAGAGUGUAGGUGACUUGUUUAC---------ACAGCUUU
R.Norvegicus ENSRNOT00000001916       UGUGUUAUUUCAGAGUGUAGAUGACUUGUUU----------ACAGCUUU
```

Rfam ID	Score	Energy	Base P	Poisson P	Org P	Start	End
hsa-miR-30c	15.5003	-25.65	7.662320e-02	7.376120e-02	2.853760e-05	643	669

B

Name	Sites
H. Sapiens	UUUUGGAGUGUAGGUGAC UUGUUUACU
M. Mulatta	UUUUGGAGUGUAGGUGAC UUGUUUACU
B. Taurus	UUUUAGAGUGUAGAUGAC UUGUUUACU
C. Familiaris	----------------------------
M. Musculus	UUUCAGAGUGUAGGUGAC UUGUUUAC–
R. Norvegicus	UUUCAGAGUGUAGAUGAC UUGUUU---

Supplementary Figure S2.

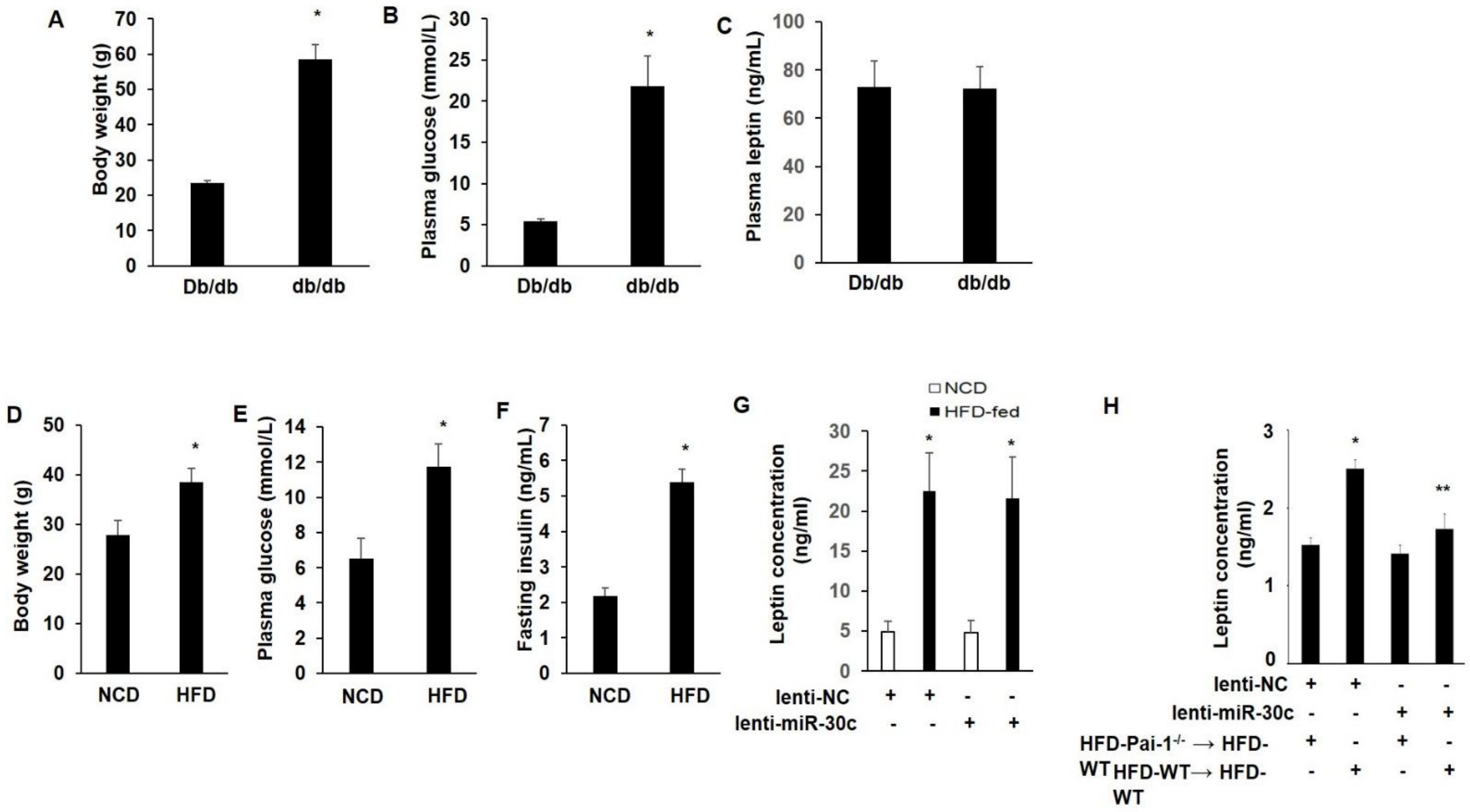

Supplement: Supplementary Information [file srep36687-s1.pdf]
